# Expression of heat-resistant β-glucosidase in *Escherichia coli* and its application in the production of gardenia blue

**DOI:** 10.1016/j.synbio.2021.08.002

**Published:** 2021-08-24

**Authors:** Wenxi Li, Jielin Li, Ying Xu, Yan Huang, Shuqi Xu, Zirui Ou, Xiaoli Long, Xinyu Li, Xinyu Liu, Zening Xiao, Jiaqi Huang, Weizhao Chen

**Affiliations:** a College of Life Sciences and Oceanography, Shenzhen University, Shenzhen, 518060, China; b College of Materials Science and Engineering, Shenzhen University, Shenzhen, 518060, China; c Health Science Center School of Biomedical Engineering, Shenzhen University, Shenzhen, 518060, China; d College of Mechatronics and Control Engineering, Shenzhen University, Shenzhen, 518060, China; e Shenzhen Key Laboratory for Microbial Gene Engineering, College of Life Sciences and Oceanography, Shenzhen University, Shenzhen, 518060, China

**Keywords:** β-glucosidase, Gardenia blue, Geniposide, Orthogonal experiment, bglA, β-glucosidase, SDS-PAGE, sodium dodecyl sulfate-polyacrylamide gel electrophoresis, BP-NN, backpropagation neural network

## Abstract

Gardenia blue is a natural blue pigment that is environmentally friendly, non-toxic, and stable. The hydrolysis of geniposide, catalyzed by β-glucosidase, is a critical step in the production process of gardenia blue. However, β-glucosidase is not resistant to high temperatures, limiting the production of gardenia blue. In this study, we investigated the effectiveness of a heat-resistant glucosidase obtained from *Thermotoga maritima* in the production of gardenia blue. The enzyme exhibited a maximum activity of 10.60 U/mL at 90 °C. Single-factor and orthogonal analyses showed that exogenously expressed heat-resistant glucosidase reacted with 470.3 μg/mL geniposide and 13.5 μg/mL glycine at 94.2 °C, producing a maximum yield of 26.2857 μg/mL of gardenia blue after 156.6 min. When applied to the dyeing of denim, gardenia blue produced by this method yielded excellent results; the best color-fastness was achieved when an iron ion mordant was used. This study revealed the feasibility and application potential of microbial production of gardenia blue.

## 1 Introduction

Plant dyes have had an essential place in the human history of exploration of color. In contrast to synthetic dyes, which can cause serious adverse effects in humans, they are generally environmentally friendly and non-toxic. Moreover, numerous harmful substances are generated in the production processes of synthetic dyes. Blue dyes are the most challenging to obtain because there are few natural sources [1]. Most natural blue pigments, such as phycocyanin and anthocyanin, have drawbacks, including poor stability and high cost for phycocyanin and color instability for anthocyanin. Therefore, these pigments require a specific pH environment, limiting their industrial production [2].

Gardenia blue, a natural dark blue pigment, has been found to have the same coloring capacity as synthetic dyes. It has a significantly higher stability than other natural dyes. The raw material for the pigment is sourced from *Gardenia jasminoides* [3], a member of the cedar family; its fruit contains geniposide, which decomposes into genipin through the action of β-glucosidase (bglA). Genipin reacts spontaneously with amino acids, peptides, or proteins to generate gardenia blue [4,5]. The most significant advantage of gardenia blue over synthetic dyes is that it is environmentally friendly.

Indigo blue, the most commonly used dye in the denim industry, has no affinity for textile fibers and requires reduction using reducing agents under specific alkaline conditions to be adsorbed onto textiles before it is oxidized to obtain the blue color. The most commonly used reducing agent for this purpose is sodium bisulfite; however, the sulfur compounds produced during the dyeing process can give rise to several problems, including sewer corrosion [6]. Moreover, the indigo dyeing process leads to the production of wastewater containing strong alkali, organic matter, and pigments. In contrast to indigo, gardenia blue can be used in the dyeing industry in an environmentally friendly manner owing to its suitable water solubility and stability [7]. Additionally, gardenia blue and the materials used in its production are harmless to the human body [4]. Moreover, geniposide has been reported to have anticancer properties [8]. Based on these properties, this pigment has been used as a food additive.

**Fig. 1:**
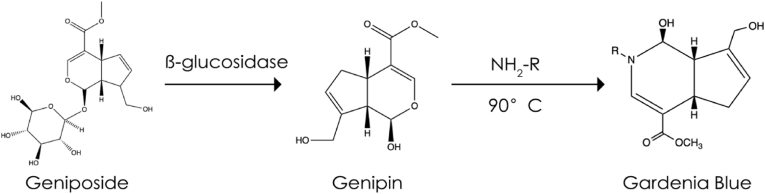
Production process of gardenia blue.

There are two common techniques for preparing gardenia blue: one-step and two-step methods. The one-step method involves direct microbial fermentation, hydrolysis, and pigment synthesis in the same container. Gardenia blue produced by this method is of low quality and has a dull color because of temperature limitations. The two-step method involves geniposide hydrolysis under the action of bglA to obtain genipin, which is subsequently reacted with an amino acid in high-temperature conditions to produce gardenia blue. Gardenia blue produced by this method is of high quality and yield. However, this two-step operation significantly increases the production time and labor costs, hindering industrial mass production. This is because bglA, which hydrolyzes the β-d-glycosidic end bond in geniposide to generate a glucose molecule and genipin, has poor thermal stability and other limitations. As a consequence, it is impossible to effectively combine the two steps of the reaction at high temperatures, making rapid and high-quality gardenia blue production challenging to achieve (Fig. 1).

The key to solving this problem lies in finding a highly heat-stable enzyme. The most reported thermophilic bacteria are *Thermotoga maritima* and its close relatives, which have an optimum growth temperature of 80 °C. BglA produced by these bacteria is the most thermotolerant glucosidase reported to date. Owing to its thermal stability, this enzyme meets the requirements for extreme industrial production conditions [9] and can maintain strong activity in such conditions.

In this study, we evaluated the recombinant expression of heat-resistant bglA obtained from *T. maritima*. Furthermore, we investigated the most appropriate formulation for gardenia blue and verified its dyeing effect with the aim to provide a theoretical basis for the efficient industrial production of gardenia blue.

## 2 Materials and methods

### 2.1 Plasmids and strains

The reagents, strains, and synthetic plasmids used in this study are listed and described in Supplementary Table S1. The plasmids were chemically transformed into *Escherichia coli* BL21 cells (Table 1). After the cells were thawed on ice, a plasmid solution (50 ng/mL) was added, and the mixture was incubated on ice for 30 min, heat-stimulated at 42 °C for 45 s, and left on ice for 3 min. The bacterial broth was incubated in 500 μL of antibacterial-free LB medium (Takara, Beijing, China) at 37 °C for 30 min and then inoculated onto LB agar plates containing 100 μg/mL ampicillin to screen the successfully transformed cells. Transformed strains were cultured in LB medium containing ampicillin at 37 °C [10].

**Table 1:** *E. coli* strain and plasmid used in this study.

Name of strain or plasmid	Genetic characteristics	Source
*E. coli* BL21	*F-, ompT, hsdSB (rB-mB-), gal, dcm*	Takara
pGEX-4T-H-beta-glucodidase	*ampR*	GenScript

### 2.2 Expression of bglA

For bglA expression, 0.6 mM isopropyl β-d-1-thiogalactopyranoside was added to bacterial broth with an OD_600_ of 0.4–0.6. The broth was then incubated at 37 °C for 6 h under shaking at 220 rpm. To obtain bglA, the bacterial broth was transferred into centrifuge tubes (1 mL), frozen in liquid nitrogen, and thawed in a water bath at 45 °C. This process was repeated five to six times until the bacteria were completely lysed. Next, the broth was centrifuged at 10,000×*g* , and the supernatant was collected into a new tube and stored at −20 °C until use. The lysates were analyzed by sodium dodecyl sulfate-polyacrylamide gel electrophoresis (SDS-PAGE).

### 2.3 BglA enzyme activity measurement

Enzyme activity was assayed using a β-Glucosidase (β-GC) activity kit (Solarbio, Beijing, China), according to the manufacturer's instructions. This assay is based on the principle that bglA decomposes p-nitrophenyl-β-glucopyranoside to p-nitrophenol, which has a maximum absorption peak at 400 nm. The unit enzyme activity per unit volume of bglA (U/mL) is defined as the amount of 1 nmol p-nitrophenol produced per unit volume per minute. The formula for calculating the enzyme activity is shown below.

$$\text{U}\left(/\text{mL}\right)=\frac{\text{D}\times 10^{3}\times \left(\text{Δ}{\text{A}}_{1}-\text{Δ}{\text{A}}_{2}\right)\times {\text{V}}_{\text{t}}}{\text{e}\times {\text{V}}_{\text{s}}\times \text{d}}$$

D : dilution times.

$\text{Δ}{\text{A}}_{1}$ : change of absorbance value of sample
$\text{Δ}{\text{A}}_{2}$ : change of absorbance value of blank control
${\text{V}}_{\text{t}}$ : total reaction system, mL
e : molar absorption coefficient
${\text{V}}_{\text{s}}$ : volume of enzyme liquid, mL
d : cuvette light position, 1 cmV

### 2.4 Production of pigments

To obtain a high-quality pigment, we investigated the most appropriate formulation for gardenia blue and optimized the amounts and types of substrate and amino acid.

A standard curve was constructed using a gardenia blue standard of 0.1 g/mL that was diluted to 1, 2, 3, 4, 5, and 6 μg/mL, and the OD_595_ was measured using a spectrophotometer.

To determine the most appropriate amino acid, 400 μL of 20 mg/mL geniposide solution and 50 μL of recombinant rough extraction protein solution were added into a 1-mL reaction tube, to which 10 μL of 0.1 mol/L glycine, l-lysine, methionine, d-glutamic acid, l-phenylalanine, l-tryptophan, or aspartic acid solutions were added.

To determine the optimal amount of geniposide, 10 μL of 140 mg/mL glycine solution, 50 μL of recombinant rough extraction protein solution, and a gradient volume of 20 mg/mL of geniposide solution (120, 160, 200, 240, 280, 320, 360, 400, 440, 480, 520, 560, or 600 μL) were added into a 1-mL reaction tube.

To determine the optimal amino acid dosage, 400 μL of 20 mg/mL geniposide solution, 50 μL of recombinant protein solution, and a gradient volume of 140 mg/mL glycine solution (1, 3, 5, 7, 9, 10, 11, 12, 13, 15, or 17 μL) were added to a 1-mL reaction tube.

All reactions were carried out at 90 °C for 3 h, after which the OD_595_ was measured spectrophotometrically.

### 2.5 Orthogonal test

The orthogonal test was conducted based on a single-factor test. The parameters for the orthogonal tests are shown in Table 2.

**Table 2:** Orthogonal test parameters.

Level	Temperature (°C)	Geniposide volume (g/L)	Glycine volume (μg/mL)	Time (min)
1	40	240	2	5
2	50	280	4	45
3	60	320	6	20
4	70	360	8	30
5	80	400	10	60
6	90	440	12	120
7	100	480	14	180

### 2.6 Backpropagation neural network (BP-NN) model

A BP-NN, with temperature, geniposide dose, glycine dose, and time as input variables and gardenia blue yield as the output variable, was established using 49 sample datasets from the orthogonal test. The main structure of the BP-NN model consisted of three layers: input, implicit, and output.

The model selected 43 sets of samples from the mixed-level test samples as training samples and the remaining six sets as test samples. The number of training iterations was set to 2,000, and the error target was set to 1 × 10^−4^. Subsequently, the samples were modeled using ‘tansig’ (S-type tangent function) for the function from the input layer to the implicit layer, and ‘purelin’ (pure linear function). ‘traingdx’ was selected as the training function.

We used cross-validation to predict different numbers of neurons in the hidden layer of the prediction model and compared the errors to determine the optimal number of neurons. To determine the simulation performance and generalization ability of the network, the independent variables of the test samples were input into the model, and the predicted values were obtained after simulation. The predicted and actual values were compared.

After the BP-NN was successfully modeled, the mapping relationship between input and output, as captured by the BP-NN, was used as the fitness function of the genetic algorithm. Then, the range of level variation for the four influencing factors (search space) was determined based on the training data samples, and 20 combinations were randomly selected as the initial cluster in the search space after endless selection, crossover, and variation operations until the termination condition was satisfied. The number of variables was set at 4; the upper and lower limits of the variables were [40 240 2 5 0.2] and [100 480 14 180]. The initial population was set at 20, double-precision was selected as the population type, Roulette was selected as the population selection method, single-point crossover was selected as the crossover method, the crossover probability was set at 0.75, the variation probability was set at 0.05, the evolutionary generation was set at 100, and the other parameters were kept as default.

### 2.7 Gardenia blue dyeing

After obtaining highly concentrated gardenia blue pigment, we verified its dyeing effect on denim textiles (Shenzhen, China) in different conditions.

The direct dyeing scheme was as follows: a water bath was heated to 40 °C, and a white denim cloth and the gardenia blue pigment were added to the water bath at bath ratios of 10:1, 30:1, and 50:1. Then, the water bath was heated to 60 °C at a rate of 2 °C/min, and this temperature was maintained for 10 min. Then, NaCl (2.3%) was added to the water bath according to the volume of the reaction system. The reaction system was maintained at 60 °C for 30 min for continuation of the dyeing process. Finally, the textile was washed with deionized water at room temperature (20–25 °C) and dried.

For the mordant dyeing method, iron ions, aluminum ions, cerium ions, or lanthanum ions were used to prepare different dyeing media. Dyeing periods of 10, 20, 30, 40, 50, and 60 min and dyeing temperatures of 30, 40, 50, 60, and 70 °C, and 80 °C were tested.

## 3 Results

### 3.1 Heat-resistant bglA expression

**Fig. 2:**
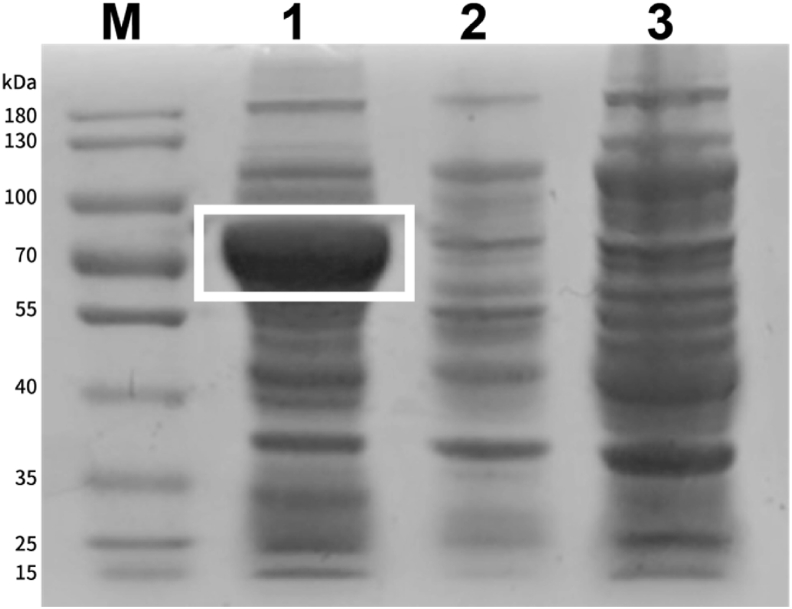
SDS-PAGE of the expressed protein. Lane 1: induced bacterial broth, lane 2: non-induced bacterial broth, lane 3: BL21 broth, lane M: pre-stained protein ladder. Bands marked with white boxes represent β-glucosidase expressed by induction.

To express the highly heat-resistant bglA in *E. coli* BL21, a plasmid harboring *bglA* was constructed and transformed into *E. coli* BL21 cells. The expressed protein was subjected to SDS-PAGE. As expected, strong expression of a protein of approximately 80 kDa was observed (Fig. 2).

### 3.2 BglA activity

**Fig. 3:**
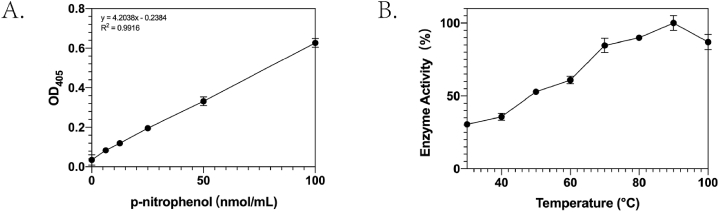
A. Standard p-nitrophenol curve. B. Enzyme activity–temperature curve for β-glucosidase.

The unit enzyme activity per unit volume of bglA (U/mL) was defined as 1 nmol of p-nitrophenol produced per min per unit volume. First, a standard curve was constructed, as shown in Fig. 3. Subsequently, enzyme activity–temperature and enzyme activity–time curves were plotted. Within a reaction time of 30 min, the enzyme activity–temperature curve showed that the activity of bglA tended to increase and then decrease with increases in temperature from 30 °C to 100 °C. The optimal activity (10.60 U/mL) was at 90 °C and bglA activity was relatively low at room temperature (Fig. 3).

### 3.3 Structural analysis of bglA

A homology-based model of the heat-resistant bglA was generated using the SWISS-MODEL program. To this end, five proteins of known structure with significant amino acid sequence similarity to bglA were selected, including family 1 bglA from *T. maritima* I (1w3j.1.A), family 1A bglA from *Thermotoga neapolitana*, mutant E349A (5idi.1.A), bglB (4hz6.1.A), *Halothermothrix orenii* bglA A (4ptv.2.A), M3-mutant bgl6 (5gnz.1.A), and heat-resistant bglA. The degrees of sequence similarity between bglA and these proteins were 73% (1w3j.1.A), 71% (5idi.1.A), 61% (4hz6.1.A), 61% (4ptv.2.A), and 61% (5gnz.1.A). A series of heat-resistant bglA models were obtained using each homologous protein as a template, and the model with the highest degree of sequence identity (73%) was selected as the final template.

**Fig. 4:**
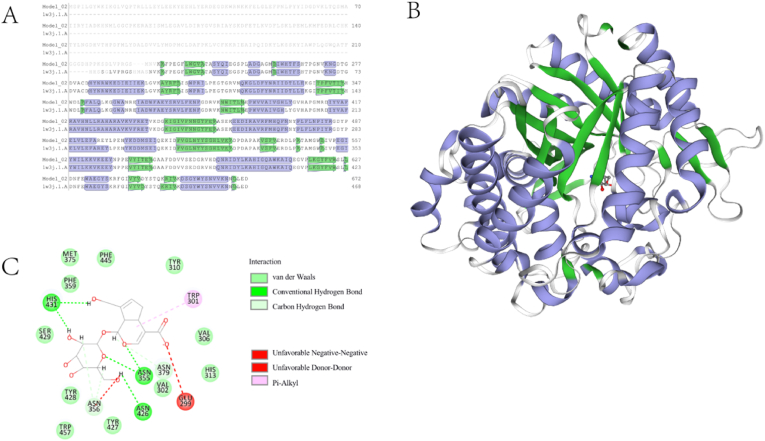
A. Amino acid sequence comparison of bglA and homology models. The purple and green structures represent the α-helix and β-fold, respectively. B. BglA homology model. C. Interaction of amino acid residues in the active centers of bglA with geniposide. (For interpretation of the references to color in this figure legend, the reader is referred to the Web version of this article.)

Molecular docking of bglA and geniposide was performed using Biovia Discovery Studio (2019, BIOVIA, San Diego, CA, US), and the positions of the vital active sites of the enzyme were determined. The top five amino acid residues that generated hydrogen bonds were ASN246, GLU166, ASN223, HIS298, and SER296, and the top five amino acid residues that generated hydrophobic interactions were TRP324, VAL173, TRP406, HIS180, and HIS298 (Fig. 4).

### 3.4 The most appropriate formula for the synthesis of gardenia blue

**Fig. 5:**
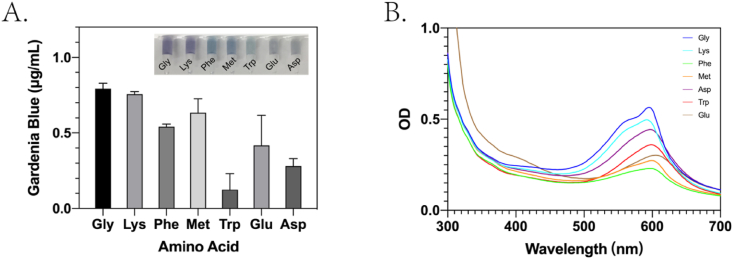
A. Gardenia blue pigments produced in the presence of different amino acids. B. Full wavelength scan of gardenia blue pigments produced using different amino acids. (For interpretation of the references to color in this figure legend, the reader is referred to the Web version of this article.)

From the graph (Fig. 5A) of the reaction of geniposide (20 mg/mL) and the different amino acids (0.1 mol/L) that constitute bglA, glycine was found to have the most translucent purple-blue color. After 3 h of reaction, we verified the absorption wavelength of the highest peak, which was determined by comparing the full wavelength scan of the reaction solution containing the different amino acids; it was then used to select the best color-forming amino acid based on OD_595_ measurement for subsequent experiments. Glycine exhibited the highest OD_595_ absorption peak and was therefore selected for use in subsequent experiments (Fig. 5B).

**Fig. 6:**
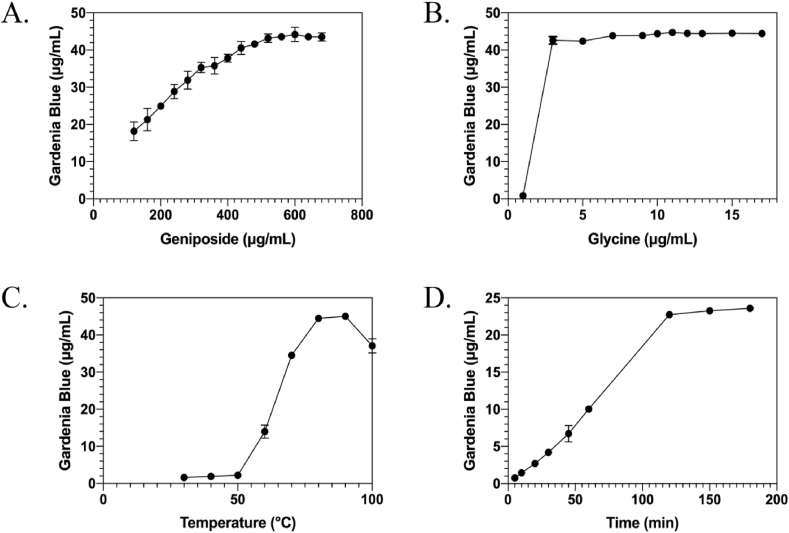
A. Gardenia blue yield–geniposide concentration curve. B. Gardenia blue yield–glycine concentration curve. C. Gardenia blue yield–temperature curve. D. Gardenia blue yield–time curve.

The gardenia blue yield increased with increasing geniposide concentration (Fig. 6A). However, no significant increase in yield was observed after a geniposide volume of 440 μL. The data were converted to mass (mg), i.e., the amount of substance (mol/mL), and concentrations of 22 μmol/mL and 18.65 μmol/mL were obtained for geniposide and glycine, respectively, yielding a ratio of 1.18:1. The glycine-gardenia blue yield curve rose sharply in the 0–6-μL interval and gradually rose to a plateau at 11 μL (Fig. 6B). The optimal temperature for gardenia blue production was found to be between 80 °C and 90 °C (Fig. 6C). Gardenia blue yield increased steadily over time, then decreased, and gradually entered a plateau phase after 120 min (Fig. 6D).

The orthogonal test results are shown in Table 3. The order of the magnitude of the effects of the four factors evaluated on gardenia blue pigment reaction, as obtained through polar difference analysis, was reaction time > reaction temperature > glycine dosage > geniposide dosage.

**Table 3:** Orthogonal test and range analysis results.

No.	Temperature (°C)	Geniposide volume (μg/mL)	Glycine volume (μg/mL)	Time (min)	Gardenia blue yield (μg/mL)
1	40	240	2	5	0.3594
2	40	280	4	45	0.5397
3	40	320	6	20	0.3821
4	40	360	8	30	0.4689
5	40	400	10	60	0.6185
6	40	440	12	120	1.0939
7	40	480	14	180	1.1687
8	50	240	4	20	0.3407
9	50	280	6	30	0.3767
10	50	320	8	60	0.5504
11	50	360	10	120	1.0579
12	50	400	12	180	1.4599
13	50	440	14	5	0.3821
14	50	480	2	45	0.4782
15	60	240	6	60	0.9764
16	60	280	8	120	1.9006
17	60	320	10	180	2.8435
18	60	360	12	5	0.3687
19	60	400	14	45	0.9110
20	60	440	2	20	0.4769
21	60	480	4	30	0.6559
22	70	240	8	180	14.0623
23	70	280	10	5	0.4221
24	70	320	12	45	1.6282
25	70	360	14	20	0.8335
26	70	400	2	30	1.0766
27	70	440	4	60	2.0489
28	70	480	6	120	4.7601
29	80	240	10	45	2.3413
30	80	280	12	20	1.0899
31	80	320	14	30	1.5226
32	80	360	2	60	2.7393
33	80	400	4	120	7.2509
34	80	440	6	180	20.9378
35	80	480	8	5	0.4582
36	90	240	12	30	2.5003
37	90	280	14	60	5.9153
38	90	320	2	120	9.7377
39	90	360	4	180	21.7658
40	90	400	6	5	0.6812
41	90	440	8	45	4.1444
42	90	480	10	20	2.0208
43	100	240	14	120	10.4910
44	100	280	2	180	20.3688
45	100	320	4	5	0.5811
46	100	360	6	45	4.6469
47	100	400	8	20	1.9807
48	100	440	10	30	3.2509
49	100	480	12	60	7.9320
K1	0.6616	4.4388	5.0338	0.4647	
K2	4.6458	4.3733	4.7404	2.0985	
K3	1.1619	2.4636	4.6802	1.0178	
K4	3.5474	4.5544	3.3665	1.4074	
K5	5.1914	1.9970	1.7936	2.9687	
K6	6.6808	4.6193	2.2961	5.1846	
K7	7.0359	2.4963	3.0320	11.8010	
R	6.3743	2.6223	3.2403	11.3363	

**Fig. 7:**
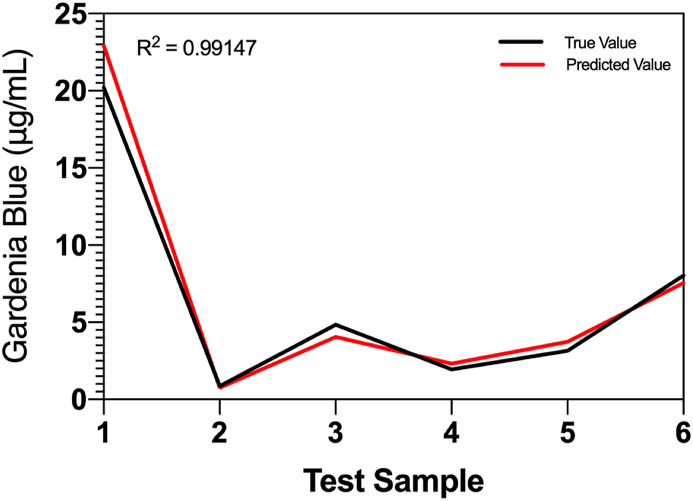
Comparison of predicted and actual values.

The results showed that the mean-squared error of the prediction value was minimized when the number of hidden layer nodes was eight. Meanwhile, the mean square error of the network reached the target value of 1 × 10^−4^ after seven iterations, and the performance of the network was stable and accurate (Fig. 7).

To determine the simulation performance and generalization capacity of the network, the independent variables of the test samples were input into the model, and predicted values were obtained after simulation and compared with actual values. The error curve in Fig. 7 shows that the error between the predicted and actual values was insignificant. The value of the coefficient of determination was high (0.99147), indicating a very high degree of agreement. Therefore, the established BP-NN model was found to be stable, with a high generalization capacity, and could be used to estimate gardenia blue yield under different preparation conditions.

After successfully modeling the BP-NN, the mapping relationship between input and output, as captured by the BP-NN, was used as a fitness function for the genetic algorithm to determine the optimal conditions for gardenia blue preparation. In a 1-mL system, by reacting 470.3 μg/mL geniposide solution with 13.5 μg/mL glycine solution for 156.6 min at 94.2 °C, the highest gardenia blue yield of 26.2857 μg/mL was achieved.

### 3.5 Dyeing effect of gardenia blue

The gardenia blue pigment was applied in the dyeing of cotton fabrics to investigate its actual dyeing effect.

**Fig. 8:**
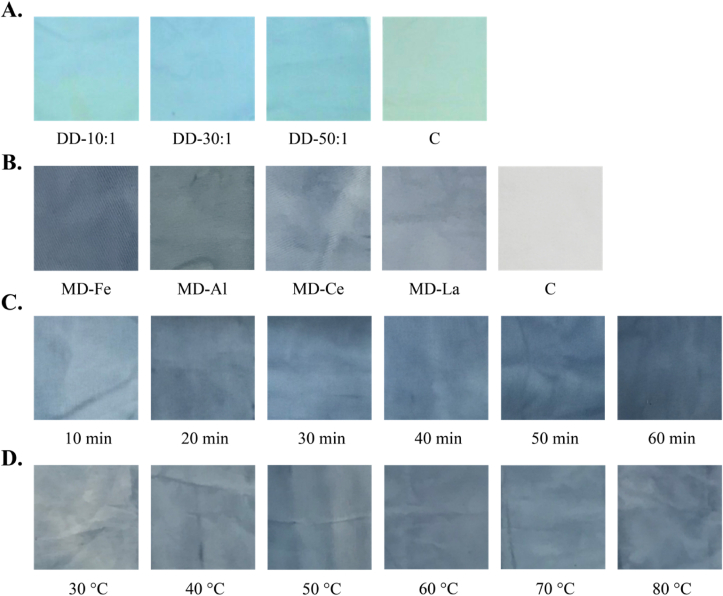
A. The direct dyeing (DD) method was used to dye denim at bath ratios of 10:1, 30:1, and 50:1. The blank control was treated without a dyeing agent. B. The mordant dyeing (MD) method was used to dye denim, with iron, aluminum, cerium, and lanthanum ions as mordants. The blank was treated without a dyeing agent. C. The MD method was used to dye denim with treatment times of 10, 20, 30, 40, 50, and 60 min. D. The MD method was used to dye denim at temperatures of 30, 40, 50, 60, 70, and 80 °C.

First, cotton fabrics dyed using direct and mordant dyeing methods were compared. For the direct method, the blue color of the dyed fabric deepened with increasing bath ratio. Fabrics dyed by the direct dyeing method were sky blue. In contrast, fabrics dyed by the mordant dyeing method had an indigo-like color. For the medium dyeing method, different factors, including different ions, dyeing temperatures, and dyeing times, were explored. The most uniform fabric color was obtained by iron ion-mediated dyeing. The optimal dye color was obtained at a dyeing time of 60 min and a dyeing temperature of 60 °C (Fig. 8).

To verify the color-fastness of the gardenia blue produced, the fabric was sent to the national testing center after mordanting with different metal ions (the control group was the “no metal ion-dyed fabric” group). Color-fastness was tested according to national standards (GB/T 3922-2013, GB/T 5713-2013, GB/T 3921-2008) and graded from 1 (worst) to 5 (best). The results are shown in Table 4. From a comprehensive perspective, the samples for which iron ions were used as a mordant exhibited the best color-fastness.

**Table 4:** Color-fastness of the samples.

		Control	Al	Fe	Ce	La
Color fastness to perspiration	Color change-acid	4	3–4	3	2–3	2
	Color staining-acid	3	3–4	3–4	2–3	2–3
	Color change-alkaline	4	3–4	3–4	2–3	2
	Color staining-alkaline	3	3	3–4	2–3	2
Color fastness to water	Color change	3–4	3–4	3	2–3	3
	Color staining	4	3	4	3–4	3
Color fastness to washing	Color change	1	1	1	1–2	1
	Color staining	4–5	4–5	4–5	4–5	4–5

## 4 Discussion

In this study, thermotolerant bglA was successfully produced in *E. coli*, and the enzyme was found to exhibit optimal activity (10.60 U/mL) at 90 °C. Recombinant bglA is a typical thermophilic enzyme with high thermal stability and optimal enzyme activity above 70 °C. However, as thermophilic enzyme-coding genes are mostly found in archaea, their codon usage rates are significantly different from that of the model bacterium *E. coli*. Therefore, the exogenous expression and purification of thermophilic enzymes are challenging. In this study, *bglA* was codon-optimized for efficient expression in *E. coli* [11]. However, there remains a need to improve the expression of the thermophilic enzyme in the heterologous host by introducing homologous transcription factors to improve enzyme yield [12].

Homology modeling of the heat-resistant bglA clearly showed that it displayed a typical TIM barrel structure, consisting of a peptide backbone with eight outer α-helices and eight inner parallel β-chains. The TIM barrel structure is common in known proteins, and the active sites of enzymes with this structure are generally located at the C-terminus of the β-chain. The salt bridge formed inside the β-chain is thought to contribute to the stability of the whole protein structure [13]. Comparison of the structure of heat-resistant bglA with those of bglB and bgl6 showed that the main structural differences were concentrated in the outer α-helix. It can be concluded that in the bglA enzyme family, the inner β-chain barrel is relatively conserved. The diversity of the outer α-helix and α,β-linkage leads to different enzyme properties, one of which is heat resistance.

With respect to gardenia blue preparation, many studies have reported the production of genipin by using bglA to hydrolyze geniposide. These studies have led to improvements in the hydrolysis efficiency of bglA by discovering new enzymes [14], mutations [15], and enzyme immobilization techniques [16]; however, the fact that the reaction between genipin and amino acids must occur at a high temperature is often neglected. For the industrial production of gardenia blue, one-step production is more cost-effective than two-step production. The present study addressed problems encountered in the production of gardenia blue and offered a solution whereby the novel heat-resistant bglA can produce gardenia blue efficiently and in large quantities. Gardenia blue was successfully produced in a single step using heat-resistant bglA, with geniposide and an amino acid as substrates. The gardenia blue yield with 470.3 μg/mL geniposide and 13.5 μg/mL glycine as substrate was considerable, with a maximum gardenia blue yield of 26.2857 μg/mL within a reaction time of 156.6 min at temperature of 94.2 °C.

Given the numerous applications of dyes in the daily lives of humans, the pollution caused by the synthetic dye chemical industry is becoming increasingly serious. The demand for natural pigments that are inherently non-toxic and harmless and have environmentally friendly production processes is increasing [17]. One of the main applications of gardenia blue is in the dyeing of fabrics. We examined the dyeing effect of gardenia blue produced by the one-step method. As the direct dyeing performance of natural pigments is relatively poor, we used different metal ion-mordant treatments to improve it. The color-fastness test results revealed that the use of iron ions as a mordant provided satisfactory color-fastness. This may be because iron ions can interact strongly with fabric fibers, and gardenia blue was adsorbed onto the fabric through ligand bonds [18]. Results of structural analysis of gardenia blue revealed that its aromatic ring can generate a six-membered ring complex following interaction with iron ions, corroborating the advantage of using iron ions as mordant [19].

The current study had a few limitations. Only glycine was selected for the experiments to optimize the synthesis of gardenia blue; optimal reaction conditions for other amino acids remain to be explored. The blue pigments produced when different amino acids are used as substrates vary (from indigo to purple colors).

A previous study [20] suggested that some peptide-containing substances, such as egg white, can replace amino acids as substrate, which would facilitate mass production of gardenia blue [21]. Moreover, gardenia blue can be used as a sensitizer in dye-sensitized solar cells [22], [23] used for photosensitized power generation.

## 5 Conclusion

A heat-resistant glucosidase-encoding gene from *T. maritima* was transformed into *E. coli* and expressed efficiently. The exogenously expressed enzyme exhibited an optimal activity of 10.60 U/mL at 90 °C. When the exogenously expressed enzyme was reacted with 470.3 μg/mL geniposide and 13.5 μg/mL glycine at 94.2 °C, a maximum yield of 26.2857 μg/mL of gardenia blue was obtained after 156.6 min. The gardenia blue pigment produced by this method showed excellent performance in the dyeing of denim, with the best color-fastness obtained when iron ions were used as a mordant.

## CRediT authorship contribution statement

**Wenxi Li:** Conceptualization, Methodology, Validation, Formal analysis, Investigation, Writing – original draft, Supervision, Project administration. **Jielin Li:** Conceptualization, Methodology, Validation, Formal analysis, Investigation, Writing – original draft, Visualization. **Ying Xu:** Methodology, Modeling, Formal analysis, Data curation, Visualization. **Shuqi Xu:** Validation, Investigation, Formal analysis. **Zirui Ou:** Validation, Investigation, Formal analysis. **Xiaoli Long:** Supervision, Project administration, Visualization. **Xinyu Li:** Methodology, Validation, Formal analysis, Investigation. **Xinyu Liu:** Methodology, Investigation. **Zening Xiao:** Investigation. **Jiaqi Huang:** Investigation. **Weizhao Chen:** Conceptualization, Methodology, Writing – review & editing, Supervision, Project administration, Funding acquisition.

## Declaration of competing interest

The authors have no known competing interests to declare.
