# Supplementary material for: Expression of heat-resistant β-glucosidase in Escherichia coli and its application in the production of gardenia blue
Source: Synth Syst Biotechnol. 2021 Aug 24;6(3):216–23. doi: 10.1016/j.synbio.2021.08.002 (PMC8390534; doi:10.1016/j.synbio.2021.08.002)
Supplement: Multimedia component 1 [file mmc1.docx]

**Table S1** Reagents used in this study.

| Reagent | Source |
| --- | --- |
| Tryptone | Sangon Biotech |
| Yeast extract | Meilunbio |
| NaCl | Xilong Scientific |
| Agar | BioFRoxx |
| IPTG | Biosharp |
| Ampicillin sodium | Sangon Biotech |
| Aspartic acid | Rhawn |
| Glycine | Shanghai yuanye Bio-Technology |
| L-lysine | Shanghai yuanye Bio-Technology |
| Methionine | Shanghai yuanye Bio-Technology |
| D-Glutamic acid | Shanghai yuanye Bio-Technology |
| L-Phenylalanine | Shanghai yuanye Bio-Technology |
| L-Tryptophan | Shanghai yuanye Bio-Technology |
| 50× TAE buffer | Shanghai yuanye Bio-Technology |
| Agargose M | Sangon Biotech |
| GoldView II Nuclear Staining Dye (5000×) | Solarbio |
| DNA loading buffer | Beyotime |
| Maker | Takara |
| SDS-PAGE precast gel | Meilunbio |
| SDS-PAGE sample loading buffer | Biosharp |
| Pre-stained protein ladder | Thermo Scientific |
| β-glucosidase (β-GC) assay kit | Solarbio |
| Geniposide | Shanghai yuanye Bio-Technology |
| Gardenia blue | Shanghai yuanye Bio-Technology |
| PBS | Leagene |
| TIANprep Mini Plasmid Kit II | Tiangen |
